# Acute Abdomen Secondary to Ileal Diverticulum: A Case Report

**DOI:** 10.7759/cureus.48693

**Published:** 2023-11-12

**Authors:** Yeudiel Suro Santos, Brando J Fematt-Rodriguez, Juan Alberto Gonzalez-Ruiz, Jose Emilio Fuentes-Hernandez, Maria Luisa Juarez-Garcia

**Affiliations:** 1 General Surgery, Instituto Mexicano del Seguro Social, Hospital General de Zona No. 33, Monterrey, MEX; 2 Surgery, Instituto Mexicano del Seguro Social, Hospital General de Zona No. 33, Monterrey, MEX

**Keywords:** small intestine disease, young adult male, diferential diagnosis, small bowel diverticulitis, jejunoileal diverticulitis

## Abstract

Small-bowel diverticulosis is rare. We report the case of a male with an acute abdomen secondary to an ileal diverticulum. A 46-year-old male complained of progressive abdominal pain over 24 hours of evolution in the left flank. On physical examination, we found abdominal pain in the left flank and mesogastrium, tenderness, and signs of peritonitis. The simple abdominal CT showed a heterogeneous tubular image in the small bowel. We performed a diagnostic laparoscopy and found a normal cecal appendix. There was no free abdominal fluid or adhesions, and the colon was without diverticula. We found a single diverticulum of 4 cm in length and 2 cm in diameter in the small intestine and therefore converted the procedure to a laparotomy. We performed a bowel resection including the diverticulum and intestinal anastomosis. The patient reported remission of symptoms after surgery.

## Introduction

Small bowel diverticulosis is rare, with a prevalence of 0.3% to 2.3%. Diverticula are herniations of the mucosa and submucosa of the intestine [1]. Most patients are asymptomatic, although the disease may present as an acute abdominal syndrome with perforation, bleeding, or intestinal occlusion [2]. We report the case of a male with an acute abdomen secondary to an ileal diverticulum.

## Case presentation

**Figure 1:**
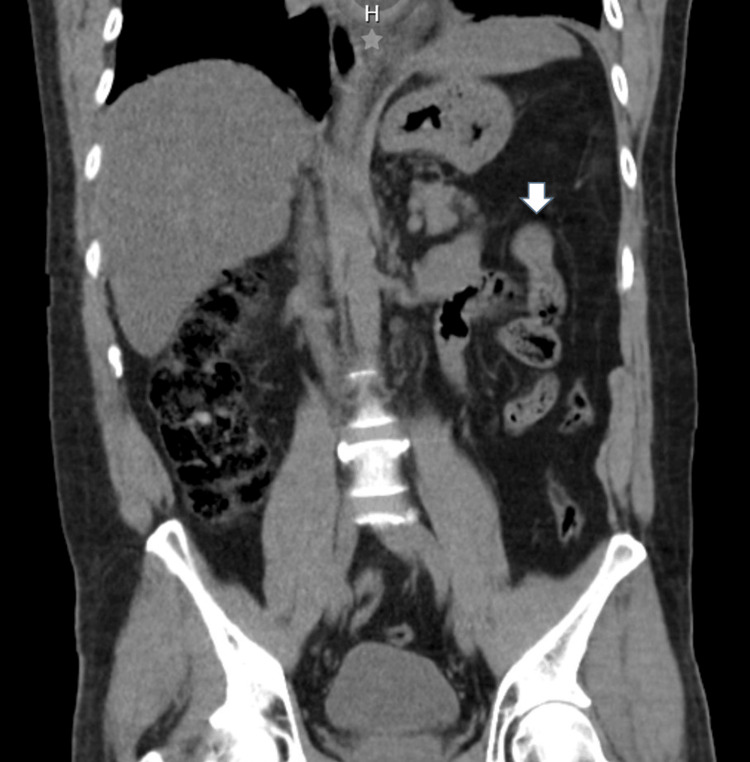
CT scan revealing heterogeneous tubular image in the small bowel (white arrow)

A 46-year-old male, without any significant past medical history, presented with the complaint of progressive abdominal pain in the left flank for the past 24 hours. He denied having fever, nausea, a change in his evacuation pattern, or vomiting. On physical examination, his vital signs were normal, but on palpation, we found abdominal pain in the left flank and mesogastrium, tenderness, and signs of peritonitis. The laboratory examination revealed a white blood cell count of 19,500 per mm3, with the rest of the test results normal. The simple abdominal CT showed a heterogeneous tubular image in the small bowel (Figure 1).

**Figure 2:**
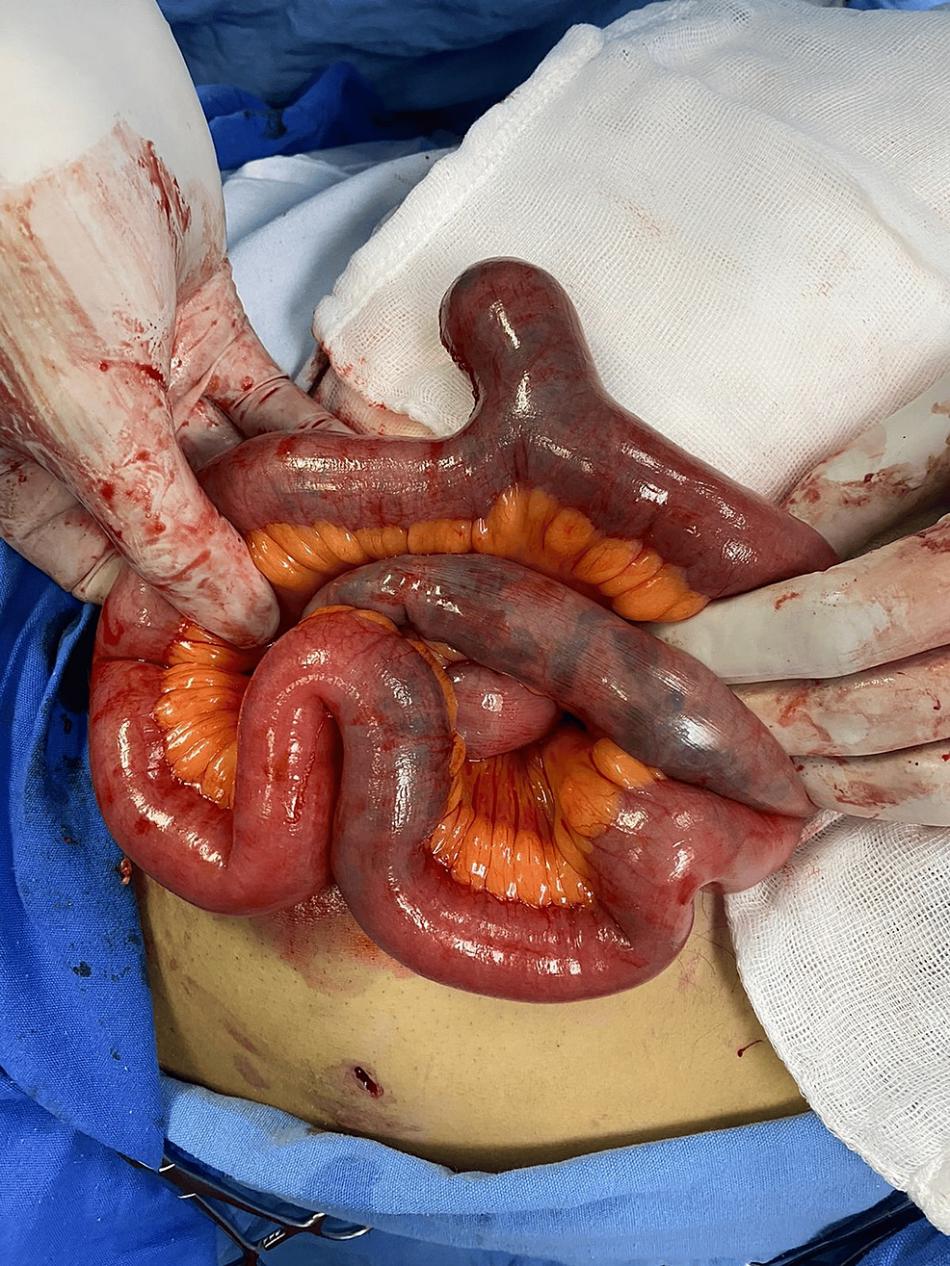
Ileal diverticulum 4 cm in length and 2 cm in diameter

We performed a diagnostic laparoscopy and found no evidence of an inflammatory process in the cecal appendix. There was no free abdominal fluid or adhesions, and there was no diverticula in the rest of the small bowel and colon. We found a single diverticulum of 4 cm in length and 2 cm in diameter in the small intestine (Figure 2).

Therefore, we converted the procedure to a laparotomy. We performed a bowel resection of 10 cm, including the diverticulum, and intestinal anastomosis. The diverticulum was located in the ileum at 280 cm from the ligament of Treitz and 100 cm from the ileocecal valve. The patient reported remission of symptoms after surgery and was discharged on the fifth postoperative day.

## Discussion

Diverticulosis of the small intestine occurs more commonly in men between the sixth and seventh decades of life [2]. In order of frequency in the small bowel, the disorder occurs mainly in the jejunum (49%), duodenum (27%), and ileum (15%) [3]. Small bowel diverticula can be congenital (Meckel’s diverticulum) or acquired. Acquired diverticula are false diverticula that usually occur in clusters and consist of mucosa and submucosa [4]. Researchers consider that pathophysiology involves intestinal dyskinesia, increased intraluminal pressure, and neuromuscular dysfunction of the small bowel [5].

Most patients are asymptomatic; the clinical presentation is nonspecific. It can be intermittent chronic abdominal pain, constipation, diarrhea, dyspepsia, intestinal occlusion, or an acute abdomen with evidence of peritonitis [6,7]. The complication rate is 10% to 20%, with hemorrhage (2% to 8%), intestinal perforation (2% to 7%), and intestinal occlusion (2% to 4.6%) [4]. Patients (39%) reported left flank pain as the most frequent pain location [1]. Imaging diagnosis is difficult; abdominal CT is the diagnostic method of choice. It can demonstrate a dilated extraluminal lesion of the bowel, wall thickening, fat striation, or free air and fluid in the case of perforation. However, the diagnosis is usually made during surgery [1,3].

There is no consensus about its treatment. If the inflammation is mild, it can be treated conservatively, with the risk of recurrence of diverticulitis. In cases of surgical management, the treatment of choice is resection of the affected intestinal segments and anastomosis [8]. Mortality rates for small bowel diverticulosis range from 0% to 5%. However, in cases of perforation, mortality is as high as 40% [9].

## Conclusions

Since small-bowel diverticulitis is rare, a delay in its diagnosis increases its morbidity and mortality. It should be among the differential diagnoses of acute abdomen that a surgeon considers. High suspicion is required for diagnosis, and a CT scan is the imaging technique of choice. When an imaging diagnosis is not possible and suspicion is high, a diagnostic laparoscopy should be performed. We recommend surgical treatment to prevent recurrences, including resection of the segment with the diverticulum and primary anastomosis.
